# Identification of iron and heme utilization genes in *Aeromonas* and their role in the colonization of the leech digestive tract

**DOI:** 10.3389/fmicb.2015.00763

**Published:** 2015-07-28

**Authors:** Michele Maltz, Barbara L. LeVarge, Joerg Graf

**Affiliations:** Department of Molecular and Cell Biology, University of ConnecticutStorrs, CT, USA

**Keywords:** iron, heme, siderophore, symbiosis, virulence factor, Aeromonads

## Abstract

It is known that many pathogens produce high-affinity iron uptake systems like siderophores and/or proteins for utilizing iron bound to heme-containing molecules, which facilitate iron-acquisition inside a host. In mutualistic digestive-tract associations, iron uptake systems have not been as well studied. We investigated the importance of two iron utilization systems within the beneficial digestive-tract association *Aeromonas veronii* and the medicinal leech, *Hirudo verbana*. Siderophores were detected in *A. veronii* using chrome azurol S. Using a mini Tn5, a transposon insertion in *viuB* generated a mutant unable to utilize iron using siderophores. The *A. veronii* genome was then searched for genes potentially involved in iron utilization bound to heme-containing molecules. A putative outer membrane heme receptor (*hgpB*) was identified with a transcriptional activator, termed *hgpR*, downstream. The *hgpB* gene was interrupted with an antibiotic resistance cassette in both the parent strain and the *viuB* mutant, yielding an *hgpB* mutant and a mutant with both iron uptake systems inactivated. *In vitro* assays indicated that *hgpB* is involved in utilizing iron bound to heme and that both iron utilization systems are important for *A. veronii* to grow in blood. *In vivo* colonization assays revealed that the ability to acquire iron from heme-containing molecules is critical for *A. veronii* to colonize the leech gut. Since iron and specifically heme utilization is important in this mutualistic relationship and has a potential role in virulence factor of other organisms, genomes from different *Aeromonas* strains (both clinical and environmental) were queried with iron utilization genes of *A. veronii*. This analysis revealed that in contrast to the siderophore utilization genes heme utilization genes are widely distributed among aeromonads. The importance of heme utilization in the colonization of the leech further confirms that symbiotic and pathogenic relationships possess similar mechanisms for interacting with animal hosts.

## Introduction

Bacteria-host interactions can manifest many different outcomes. These outcomes can range from the commonly investigated pathogenic ones, in which microbes have a negative effect on a host, to mutualistic interactions, in which both microbe and host benefit. Despite the disparity in the end result, some studies have revealed a similarity in the molecular requirements of pathogenic and beneficial relationships (Hentschel et al., 2000; Steinert et al., 2000; Falkow, 2004; Silver et al., 2007a). A better understanding of colonization factors in mutualistic symbioses can lead to a clearer perception of conserved processes underlying microbe –microbe and microbe-host interactions.

The medicinal leech, *Hirudo verbana*, is a freshwater parasite that feeds exclusively on vertebrate blood. During a single feeding, the medicinal leech can consume over five times its body weight, after which the blood is stored in the largest compartment of the digestive tract, the crop (Sawyer, 1986). The leech modifies the ingested blood meal by removing water and osmolytes from each meal (Sawyer, 1986). The complement system of the ingested blood meal remains active for some time preventing sensitive bacteria from colonizing the intraluminal fluid (ILF) (Indergand and Graf, 2000; Braschler et al., 2003). In addition, leech hemocytes, macrophage-like cells of invertebrates, infiltrate the crop and phagocytose sensitive bacteria (Silver et al., 2007b). Within ILF of the crop, the digestive-tract symbionts reside and proliferate. The symbionts require many molecular tools for successful colonization and persistence within this environment (Graf, 2006; Silver et al., 2007a; Nelson et al., 2012). These molecular tools can be critical during the first 24 h of incubation, especially the ability of *A. veronii* to lyse erythrocytes (Maltz and Graf, 2011). All of these features help to maintain the leech gut as a simple microbial community, dominated by two species, *Aeromonas veronii* and *Mucinivorans hirudinis* (Graf, 1999a; Worthen et al., 2006; Nelson et al., 2015). *A. veronii*, a Gram-negative, facultative anaerobe, is both a symbiont of *H. verbana* and a human pathogen. It can cause diseases such as septicemia, wound infections, and gastroenteritis (Janda et al., 1984, 2010; Janda and Abbott, 1998, 2010; Senderovich et al., 2008). *M. hirudinis* is Gram-negative, an obligate anaerobe and represents a new genus *Mucinivorans* in the Bacteroidetes family that was in previous studies referred to as a *Rikenella*-like bacterium (Worthen et al., 2006). The simplicity of the leech gut microbiome and the ability to genetically manipulate *A. veronii* allows one to study factors important for microbe-microbe interactions and microbe-host interactions in a naturally occurring mutualistic symbioses (Graf, 2006).

In blood, low levels of free iron are present because most iron is bound to specialized iron-binding proteins, such as transferrin in plasma or hemoglobin inside erythrocytes (Crosa, 1989; Barghouthi et al., 1991; Crosa and Walsh, 2002). Most microorganisms require iron for metabolic processes and have evolved sophisticated mechanisms to obtain protein-associated iron with in a host (Crosa, 1989; Litwin and Calderwood, 1993; Crosa and Walsh, 2002). These mechanisms can be divided into siderophore-dependent and siderophore-independent iron acquisition systems (Byers et al., 1991).

Siderophores are low-molecular weight iron chelators that microbes secrete into the environment under iron-limiting conditions. In many bacteria, the ferric uptake regulator, Fur, regulates siderophore biosynthesis (Crosa, 1989; Crosa and Walsh, 2002). Siderophores can have a very high affinity for iron, enabling microbes to obtain iron in the presence of transferrin or free iron found in the environment. Once iron is bound to the siderophore, the iron-siderophore complex associates with a siderophore receptor located on the outer membrane of cell and is transported into the cytoplasm in an ATP-dependent manner. In the cytoplasm, the iron is dissociated from the iron-siderophore complex and used for cellular processes (Crosa, 1989; Zywno et al., 1992; Crosa and Walsh, 2002). *Escherichia coli* Fes and *Vibrio cholerae* ViuB are esterases that hydrolyze the ligand, releasing iron (Butterton and Calderwood, 1994).

Some microbes also have receptors specialized for obtaining host-produced iron-containing molecules such as transferrin or heme (Byers et al., 1991). This process of iron acquisition is siderophore-independent. For example, the heme receptor has a high affinity for heme, enabling microbes to bind heme and subsequently dissociate iron from heme. In Gram-negative bacteria, a heme receptor located in the outer membrane binds to heme and heme is translocated into the cytoplasm in a TonB dependent manner (Stojiljkovic and Perkins-Balding, 2002).

Iron utilization has been shown to be an important virulence factor in several pathogenic associations, including *Haemophilus influenzae, V. cholerae, Yersinia* spp., and *Corynebacterium diphtheriae* (Litwin and Calderwood, 1993; Morton et al., 2007, 2009). However, in beneficial symbioses, only a few systems have shown iron utilization as an important colonization factor; e.g., *Rhizobium leguminosarum, Sodalis glossinidius, and Vibrio fischeri* (Nadler et al., 1990; Graf and Ruby, 2000; Hrusa et al., 2015). *Aeromonas* species have been shown to posses at least two different iron utilization mechanisms, siderophores and a receptor for heme-containing molecules (Barghouthi et al., 1989a,b). Under iron limiting conditions *Aeromonads* can produce one of two siderophores, either enterobactin or a set of four bis-catecholate siderophores named amonabactin (Telford et al., 1994). It remains unclear whether *Aeromonas* species use one or both of these iron utilization mechanisms during infection or symbiosis. In this study we determined that an *A. veronii* strain isolated from the leech crop, HM21, possess two high-affinity iron-utilization systems. Our goal was to determine whether one or both of these iron acquisition systems are important for *A. veronii* to colonize the leech digestive tract. We evaluated the importance of siderophore acquisition and heme utilization by generating mutants and double mutants and characterizing these strains. We also examined the prevalence iron uptake systems in other *Aeromonas* species.

## Materials and methods

### Bacterial strains and growth conditions

The *A. veronii* strains were cultured at 30°C and *Escherichia coli* at 37°C in Luria broth (LB) (Sambrook and Russell, 2001), or low iron medium (LIM) (Cox, 1994) or LB agar plates (15 g/l of BactoAgar). For experiments requiring low iron levels, glassware was acid washed overnight in 6 M HCl or new plastic ware was used (Cox, 1994). The concentrated 5xM9 salts were prepared by dissolving, per liter, 30 g Na_2_HPO_4_, 15 g KH_2_PO_4_, 5 g NH_4_Cl, and 2.5 g NaCl. M9-based (Sambrook and Russell, 2001) LIM was prepared by adding the following to 748 ml of Nanopure (Barnstead, Dubuque, IA) water: 200 ml 5xM9 salts, 20 ml 20% glucose, and 30 ml 10% Bacto™ casamino acids. Residual iron was removed by adding Chelex® 100 Resin (Bio-Rad, Hercules, CA) at a concentration of 100 g/l medium. The resin was stirred with the medium for 1 h. Afterwards, the medium was filtered into acid-washed glassware. The pH of the LIM was adjusted to pH 7.0 with HCl. The medium was filter sterilized (0.2 μm) and MgSO_4_ (2 ml/l) and CaCl_2_ (0.1 ml/l) were aseptically added. Chelex® 100 Resin was restored and reused as recommended by the manufacturer; used resin was stirred for 1 h in two volumes of 1 M HCl. The resin was filtered and washed in 5 volumes Nanopure water, followed a rinse with 2 volumes 1 M NaOH. After the NaOH was removed, the resin was subjected to numerous 30 min long, 5-volume washes in Nanopure water. These rinses were repeated until the resin suspension had a pH between 10 and 11, and the resin was subsequently air-dried. For LIM plates, water was replaced with agar cooled to 50°C. The growth medium was supplemented with the appropriate antibiotics at the following concentrations: ampicillin, 100 μg/ml; chloramphenicol (Cm), 1 μg/ml for *A. veronii* and 30 μg/ml for *E. coli*; kanamycin (Km), 100 μg/ml; rifampin (Rf), 100 μg/ml for selection and 10 μg/ml for maintenance; streptomycin (Sm), 100 μg/ml; and trimethoprim (Tp), 100 μg/ml.

### Generation of *A. veronii* siderophore mutants

The *A. veronii* siderophore mutants were derived from HM21R (Table 1) by conjugation with *E. coli* strain BW20767 harboring pRL27, which carries a miniTn*5*, as described previously (Larsen et al., 2002; Silver et al., 2007a,b; Maltz and Graf, 2011). Thousand nine hundred mutants were screened on CAS (chrome azurol S) (Km, Rf) agar plates for color changes of the medium surrounding the colonies. CAS is an iron-dye complex that changes color from blue to orange when iron is dissociated from CAS. CAS (Km, Rf) agar plates were prepared as previously described by Schwyn and Neilands (1987; Neilands, 1994).

**Table 1:** **Bacterial strains and plasmids**.

**Bacterial strains and plasmids**	**Characteristics**	**Source or reference**
***A. VERONII* STRAINS**		
HM21R	Parent strain, Rf ^r^	Graf, 1999a
HM21RS	Parent strain, Rf ^r^, Sm^r^	Graf, 1999a
S-497	HM21R, *viuB*::mTn*5*, Rf^r^, Km^r^	This study
S-479S	S-497, Rf^r^, Km^r^, Sm^r^	This study
H-890	HM21R, *hgpB::Cm*^r^, Rf^r^, Cm^r^, Sm^r^	This study
SH-894	S-497S, *hgpB::Cm*^r^, Rf^r^, Km^r^, Cm^r^, Sm^r^	This study
HM21TP	HM21R, Tn*7*, Rf ^r^, Tp^r^	This study
HM21C1	HM21R, Tn*7::hgpB*, Rf ^r^, Tp^r^	This study
HM21C2	HM21R, Tn*7::hgpBR*, Rf ^r^, Tp^r^	This study
SHTP	SH-894, Rf ^r^, Km^r^, Cm^r^, Sm^r^, Tp^r^	This study
SHC1	SH-894, Tn*7::hgpB*, Rf ^r^, Km^r^, Cm^r^, Sm^r^, Tp^r^	This study
SHC2	SH-894, Tn*7::hgpBR*, Rf ^r^, Km^r^, Cm^r^, Sm^r^, Tp^r^	This study
SHC3	SH-894, Tn*7::hgpB*+intergenic regions,Rf ^r^, Km^r^, Cm^r^, Sm^r^, Tp^r^	This study
SHC4	SH-894, Tn*7::hgpR*, Rf ^r^, Km^r^, Cm^r^, Sm^r^, Tp^r^	This study
***E. coli* STRAINS**		
S17-1 λ pir	Strain used for conjugation with *A. veronii*	de Lorenzo and Timmis, 1994
BW20767	Conjugation donor strain	Boccazzi et al., 2000
DH5 αλ pir	Cloning strain capable is maintaining suicide vectors	Boccazzi et al., 2000
Top10	Cloning strain; Km^r^	Invitrogen
**PLASMIDS**		
pBC SK	Cm^r^	Stratagene
pKAS46	pGP704, rpsL, Km^r^	Skorupski and Taylor, 1996
pCR2.1	lac promoter, lacZα fragment, Ap^r^, Km^r^, pUC ori, fl origin	Invitrogen
pMM1	pCR2.1, Cm^r^	This study
pMM2	pCR2.1, 1.5 kb hgpB fragment	This study
pMM3	pCR2.1 1.5 kb hgpB fragment, Cm^r^	This study
pMM4	pKAS46, 1.5 kb hgpB fragment, Cm^r^	This study
pUC18R6KT-miniTn7T	Ap^r^, R6K replicon, oriT, Tn7	Choi et al., 2005
pFTP1	Cassette vector, Ap^*r*^, Tp^r^, source of TP^r^ cassette	Choi et al., 2005
pTn*7*Tp	Ap^r^, Tp^*r*^, R6K replicon, oriT, Tn7	This study
pTnHA	Ap^r^, Tp^*r*^, R6K replicon, oriT, Tn7::hgpB	This study
pTn7C2	Ap^r^, Tp^*r*^, R6K replicon, oriT, Tn7::hgpBR	This study
pEVS104	Helper Tra, Trb, Km^r^ Ap^r^, R6K replicon;	Stabb and Ruby, 2002
pTNS2	Encodes the TnsABC+D specific transposition pathway	Choi et al., 2005
pRL27	Km^r^, modified Tn5 plasposon	Larsen et al., 2002
pBBL3	Km^r^, carries transposon insertion and flanking DNA from S-497	This study

#### EDDA inhibition assay

The mutants were tested for the ability to obtain iron in the presence of the iron chelator ethylenediamine-di (*o*-hydroxyphenlacetic acid), EDDA, by adding EDDA for a final concentration of 150 μM in LIM or LIM plates. All cultures were grown for 24 h at 30°C. Heme at a final concentration of 50 μM was added to broth containing an inhibiting concentration of EDDA (150 μM) (Crosa and Walsh, 2002).

#### Molecular characterization siderophore utilization mutant

The site of the transposon insertion was determined by extracting DNA from the mutant, digested using *EcoR*I self-ligating and electroporating the DNA into *E. coli* strain S17-1 λ *pir*. DNA was extracted using DNeasy Blood & Tissue kit (Qiagen Valencia, CA). The resulting plasmid (pBBL3) was sequenced using outward facing primers for miniTn*5* (tpnRL17–1 AACAAGCCAGGGATGTAACG and tpnRL13–2 CAGCAACACCTTCTTCACGA). A BLASTX search of the NCBI database identified presumptive homologs. The *A. veronii* genome was then searched for the presumptive homologs.

### Construction of *A. veronii* heme receptor mutants

The *A. veronii* genome was searched for a gene encoding a heme receptor by comparing the presumptive heme receptor, HgpB, from *A. hydrophila* to the deduced amino acid sequence of the genome using BLASTX (Bomar et al., 2013). A 1.5 kb fragment containing the presumptive heme receptor gene (*hgpB*) was PCR amplified from Hm21 DNA using primers hgpB1F 5′CGTATTTGACCCGA GCATC′3 and hgpB2R 5′TCTAATCATGGG ATCTCACGGC′3. The reaction mixture contained 100 ng of DNA, 5 μl PCR buffer, 1.5 mM MgCl_2_, 200 μM each dNTP, 0.2 μM each primer, and 1 U of Platinum *Taq* DNA polymerase (Invitrogen, Carlsbad, CA) in a final volume of 50 μl. The amplification conditions were as follows: (i) 5 min at 95°C; (ii) 30 cycles of 30 s at 95°C, 30 s at 60°C, and 90 s at 72°C. The PCR product was cloned into pCR®2.1 using a TA Cloning^©^ Kit (Invitrogen, Carlsbad, CA) yielding pMM2. The identity of the insert as *hgpB* was confirmed by DNA sequencing. A chloramphenicol (Cm) cassette was amplified from pBCSK+∕− (Stratagene) using the following primers with added *Sal*I restriction sites, CMF1 5′CGAGTCGACTCCAACTTT CACCATAATGA′3 and CMR1 5′CTAGTCGACGATCTCAACAGCGGTAAGAT′3. The reaction mixture was as described above except that 1 mM MgCl_2_and 0.5 μM of each primer were present in the reaction mix. The amplification conditions were the same as described above except the annealing temp was 70°C. The Cm^R^ cassette PCR product was inserted into pCR®2.1 using a TA Cloning^©^ Kit yielding pMM1. The Cm^R^ cassette was then excised from pMM1 using *Sal*I and inserted into a *Sal*I site in the middle of *hgpB* yielding pMM3. The interrupted gene was moved from pMM3 into pKAS46 using *EcoR*I yielding pMM4 (Skorupski and Taylor, 1996).

The *A. veronii hgpB* mutants were derived from HM21RS and S-497S (Table 1). HM21RS and S-497S are spontaneous streptomycin resistant mutants from HM21R and S-497, respectively. In a tri-parental mating with the helper plasmid pEVS104, the suicide plasmid pMM4 was moved into either HM21RS or S-497S yielding a heme receptor mutant (H-890) and a siderophore/heme receptor double mutant (SH-894), respectively. For the conjugations, 5 × 10^7^ CFU of each donor and 1 × 10^8^ CFU of the recipient cells were mated overnight and double cross-over events were selected for by plating on LB Sm100 μg/μl Cm1 μg/μl, because pMM4 carries an *rpsL* allele that confers dominant sensitivity to Sm. The presumptive mutants were verified with PCR using hgpB1F and hgpB2R primers, comparing the size of the PCR product amplified from the mutants to Hm21, as previously described (Rio et al., 2007).

#### Colonization assay method and blood *in vitro* assays

The ability of the mutants to colonize the crop of *H. verbana* was done as described previously (Graf, 1999b; Rio et al., 2007; Silver et al., 2007b), with following modifications. Each of the strains was added to 5 ml of sheep blood (500 CFU/ml)from which 500 μl were removed for the *in vitro* assay and the remainder was fed to a leech. During iron restoration experiments, a final concentration of 150 μM of EDDA and/or 160 μM of FeCl_3_ were added to the blood meals along with strains. The inoculated blood and the leech were incubated at room temperature for a time course of 18, 24, 42, and 72 h. The leech was sacrificed and the ILF and blood samples were plated on LB agar Rf. Differences in the colonization levels were analyzed using GraphPad Prism software. The Mann–Whitney *t*-test was performed to calculate *p*-values.

#### Genetic restoration of *A. veronii* heme utilization defect

The SH-894 double mutant was complemented using Tn*7*. A trimethoprim cassette was excised from pFTP1 with *Bam*HI (NEB), gel extracted using QIAquick Gel Extraction Kit (Qiagen), and cloned into pUC18R6KT- mini-Tn*7*T using the *Bam*HI site located inside the Tn*7*, yielding pTn*7*Tp (Choi et al., 2005). The following genes were PCR amplified from HM21 genomic DNA: *hpgB* gene, *hgpB* + *hgpR* together, and *hgpR*. The following primer sets were used for amplification: for *hpgB* amplification—HgpR 5′CACCCATTGTAA AGAATTAC′3 and HgpF 5′CAGGGCGACGACAGTGTA AAACC′3, for amplification of both genes (*hgpB* + *hgpR*)—Trans + HgpBR 5′GTAGTC GCTCGGATAGTT′3 and Trans + HgpBF 5′ATGTCATCACATCAC TGG′3, and for amplification of hpgB only- AHyFR 5′TTC AACCTGTTTGACAAGGA′3 and AhyRR 5′ACGTAA TGAGCAAGCTTTTG′3. The reaction mixture contained a final concentration of 100 ng of DNA template, 1X GoTaq® Green Master Mix (Promega), 1 μM of either primer set in a final volume of 25 μl. The amplification conditions were as follows: (i) 5 min at 95°C; (ii) 30 cycles of 30 s at 95°C, 30 s at 60°C, and 2 min at 72°C. The PCR product was cloned into pCR®2.1 using the TA Cloning^©^ Kit (Invitrogen). *hgpB* or *hgpR* or *hgpB* + *hgpR* was then digested out of pCR2.1 using *Eco*RI restriction sites and cloned into pTn*7*TP using *Eco*RI restriction sites located on the Tn*7* yielding pTn*7*C1 and pTn*7*C2. Quad-parental matings were performed to conjugally transfer the transposon containing no insert, pTn*7*TP; *hgpB*, pTn*7*C1; or *hgpB* and *hgpR*, pTn*7*C2; hgpR, pTN7C3 into Hm21 or SH-894 yielding HM21TP, HM21C1, SHTP, SHC1, SHC2, SHC4, respectively (Table 1 and **Figure 2A**). The two additional plasmids need for the quad-parental matings were, pEVS104, which encodes *tra* genes and pTNS2 that carries the Tn*7* transposase. The plasmid pTNS2 does not encode *tnsE*, making the transposition specific for insertion into the *glnS* region of the chromosome (Choi et al., 2005). The insertion of Tn*7* downstream of *glnS* was verified (Choi et al., 2005) using inverse PCR (Ochman et al., 1988). Genomic DNA was extracted from Hm21TP, Hm21C1, SHTP, SHC1, HM21C2, and SHC2 using MasterPure DNA Purification Kit (Epicentre Biotechnologies). Genomic DNA was digested using *Nco*I (New England Biolabs) overnight at 37°C. A self-ligation was preformed on the digests using T4 DNA ligase (New England Biolabs). PCR reaction mixture contained 2 μl ligation reaction, 1X GoTaq® Green Master Mix (Promega), 1 μM Tn*7*R and Tn*7*L (Choi et al., 2005) in a final volume of 25 μl. PCR products were sequenced as described below using Tn*7*R and Tn*7*L primers.

#### DNA sequencing and sequence analysis

DNA Sequencing and analysis was performed as previously described in Silver et al. (2007b). The DNA sequences obtained in this study were deposited in GenBank under the accession numbers HM569268 and HM569269.

#### Reverse transcription PCR (RT-PCR) and quantitative reverse transcription PCR (qRT-PCR)

For both RT-PCR and qRT-PCR, RNA was isolated using Epicentre MasterPure RNA purification kit (Epicentre Biotechnologies, Madison, WI). After RNA was isolated the TURBO DNA-free kit (Ambion, Austin, TX) was used to remove any remaining DNA. The absence of DNA was confirmed by performing the PCR amplification without the reverse transcriptase on each sample using hgpB1F and hgpB2R primers. The reaction mixture contained 1.5 μl RNA template, 1x GoTaq® Green Master Mix (Promega), 1 μM hgpB1F and hgpB2R in a final volume of 25 μl. cDNA was synthesized using SuperScript III First-strand Synthesis SuperMix (Invitrogen). The reaction mixture contained 5 μl RNA, 1 μl random hexamers, 1 μl annealing buffer, 2 μl enzyme mix, 10 μl first-strand mix, and 1 μl in a final volume of 20 μl. Following first-strand cDNA synthesis for RT-PCR, PCR was performed using GoTaq® Green Master Mix, equal amounts of cDNA was used. For both RT-PCR and qRT-PCR the primer used were: for *hpgR* (Trans.activatorR 5′CGTGCCAGGGAATCGT GATC3′, Trans.activatorF 5′AGGACATCGCTGGGT TGG3′), *hgpB* (HemeF1 5′TTGAGCTTGACC GCATCCGG3′, HemeR1 5′GGTGAAGTGGAGAAC CTGCTGC3′), and *rpoB* (rpoBF 5′TTATCGTCT CCAGCTGCACCG3′, rpoBR 5′TGCTGGCAG TTTGCGACGAC3′). For qRT-PCR a reaction mix was set up using Sso Advanced™ Universal SYBR® Green Supermix and the CFX96-Real Time system (Bio-Rad Laboratories, Hercules, CA). The reaction mix contained 5 μl Sso Advanced™ Universal SYBR® Green Supermix, 1.5 μl forward primer, 1.5 reverse primer, 1 μl cDNA and 1 μl H_2_O for a final volume of 10 μl. Quantitative measurements were performed on biological samples in triplicate and results were normalized to HM21 housekeeping gene *rpoB*.

#### Genomic analysis of other *Aeromonas* strains

Genomes were downloaded from NCBI and annotated with RAST as previously described (Aziz et al., 2008; Colston et al., 2014) and the iron utilization genes from *A. veronii* Hm21, amonabactin gene *amoA*, and enterobactin receptor from *A. hydrophila* CECT839^T^ were used to query the publically available genomes. The nucleotide sequences were queried using blastn (version 2.2.30+) with the word size set of 9 to increase sensitivity and requiring 75% of the query to be present in the hit.

## Results

### *A. veronii* (HM21) produces a siderophore and utilizes heme-containing molecules as an iron source

**Figure 1:**
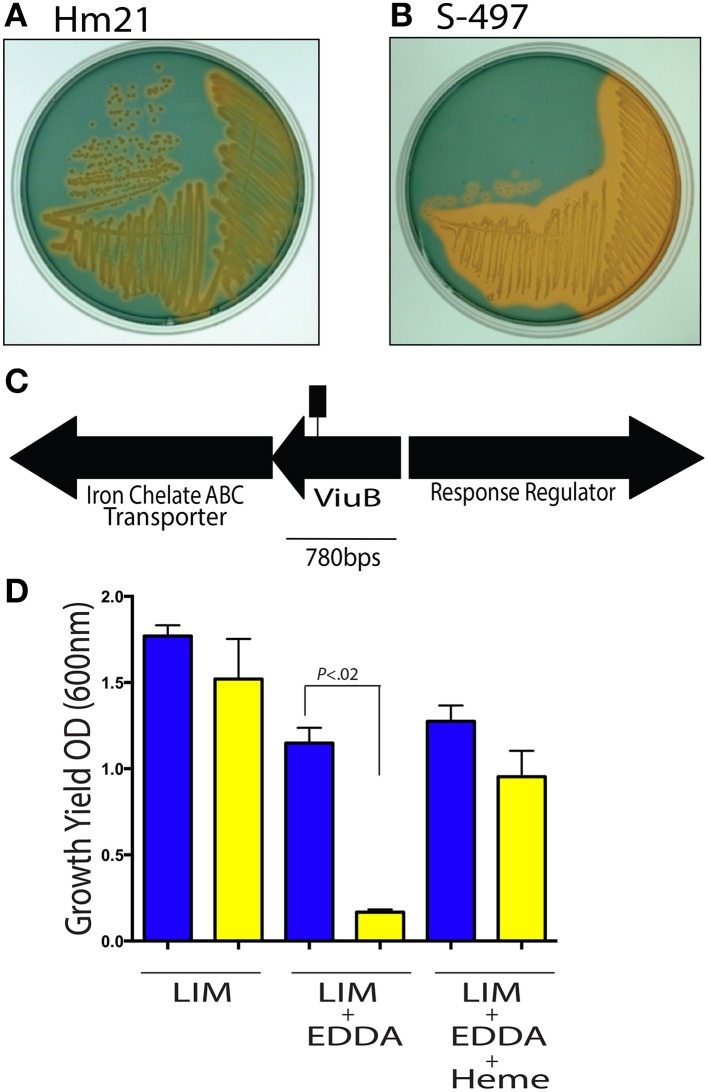
**Siderophore activity in *A. veronii***. The siderophore production of the parent strain Hm21R **(A)** and the transposon mutant S-497 **(B)** were assessed using CAS agar. The iron-CAS complex changes color from blue to orange when iron is dissociated from CAS and thus can reveal the production of a siderophore. A portion of the siderophore locus from *A. veronii*, Hm21, is shown **(C)**. The transposon insertion site is indicated by the square lollipop **(C)**. The *in vitro* LIM growth yield of HM21R (blue) and S-497 (yellow) mutant was determined by measuring the OD_600_ 24 h after inoculation in LIM, LIM (150 μM EDDA) or LIM (150 μM EDDA and 50 μg/ml hemin) **(D)**. The statistically significant difference between the parent strain and each mutant was performed using the Mann–Whitney test. This revealed HM21 possess a mechanism for utilizing iron from heme containing molecules.

Our goal was to determine if the *A. veronii* leech-isolate, HM21, requires an iron utilization system for successfully colonizing the leech gut. The ability of HM21 to produce siderophores was revealed by streaking on agar containing chrome azurol S (CAS). When siderophores have a higher affinity for ferric iron than CAS, the siderophores remove Fe^3+^ from CAS leading to a dramatic color change from deep blue to orange or yellow. The presence of an orange halo around the colonies suggested that HM21 produces siderophores that have a greater affinity for Fe^3+^ than CAS (Figure 1A). This approach was used to screen for transposon mutants with altered siderophore production phenotypes. Mutants were generated in a spontaneous Rf^R^ mutant of HM21, HM21R, using a miniTn*5* (Graf, 1999a; Larsen et al., 2002; Maltz and Graf, 2011). A total of 1900 *A. veronii* miniTn*5* mutants were screened on CAS agar for changes in the iron sequestration phenotype. This screen bared two mutants with altered siderophore production phenotypes. Mutant S-497 produced a brighter halo then the parent strain HM21R, indicative of an excessive siderophore productivity phenotype (Figures 1A,B), and mutant S-495 produced less siderophore than the parent strain (data not shown). The insertion site was identified by sequencing the flanking DNA using inverse PCR (Figure 1C). BLASTX searches of the NCBI database revealed that the deduced amino acid sequence of the inactivated gene in S-497 was 65% identical to ViuB (vibriobactin utilization protein) from *A. hydrophila*, suggesting that this mutant is unable to utilize iron bound to a siderophore since it cannot hydrolyze the ligand and release iron. In response, S-497 is iron starved and overproduces siderophores. BLASTX searches of the NCBI database revealed in S-495 the Tn inserted in the siderophore receptor. Like *Vibrio anguillarum* and other Gram-negative bacteria, the siderophore receptor in HM21 could be located within a siderophore biosynthetic operon, causing the polar effect of the Tn insertion to inactive multiple biosynthetic genes (Actis et al., 1986). For this reason we focused on S-497 in this study.

The *viuB* mutant was further characterized under iron limiting conditions by exploring *in vitro* phenotypes through measuring growth yields. In a LIM, HM21R, and S-497 reached a similar density 24 h after inoculation (Figure 1D). The effect of sequestering iron with 150 μM of the iron chelator EDDA revealed that the growth yield of S-497 was significantly lower than HM21R, showing the importance of siderophores for acquiring iron under these growth conditions. Interestingly, the addition of heme (50 μM) restored the growth yield of S-497 to that of the parent strain, suggesting S-497 can utilize heme-containing molecules in a siderophore-independent manner.

### HM21 genome contains a heme receptor

**Figure 2:**
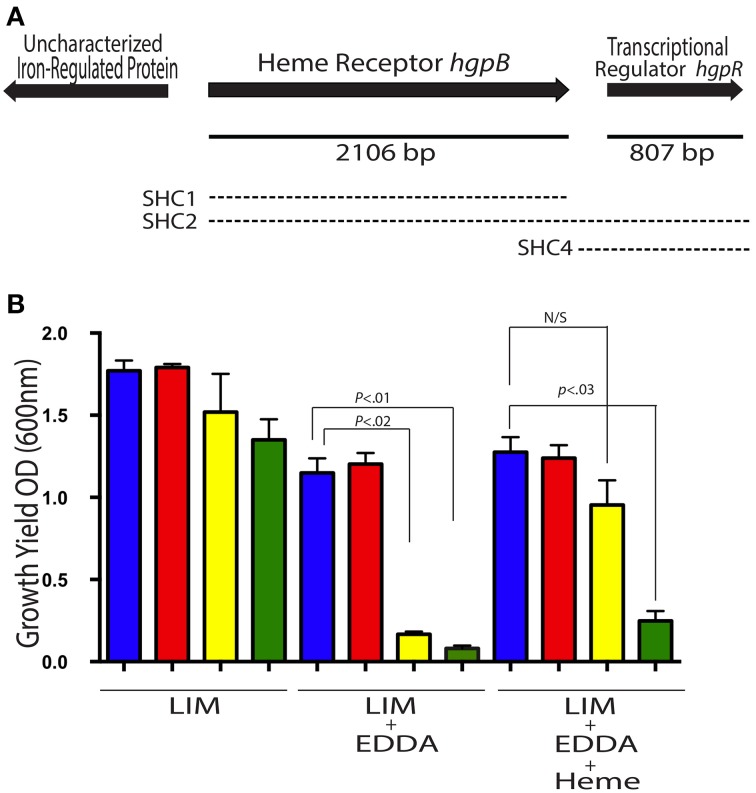
***A. veronii* possess gene for utilizing iron from heme**. The heme receptor locus from HM21 is shown **(A)**. The solid line indicates length of the gene. The three regions used for complementation with a Tn*7* are represented by the dashed lines, the upper dashed line (SHC1), the middle dashed line (SHC2), and the bottom dashed line (SHC4). **(B)** The growth yield of Hm21R (blue); H-890 (red), the *hgpB* mutant; S-497 (yellow), the siderophore mutant; and SH-894 (green), the double mutant was determined by measuring the OD_600_ 24 h after inoculation in LIM, LIM (150 μM EDDA), or LIM (150 μM EDDA and 50 μg/ml hemin). The statistically significant difference between the parent strain and each mutant was performed using a Mann–Whitney test. This revealed the requirement of the *hgpB* for utilizing hemin as an iron source in the presence of EDDA.

Our next goal was to identify the genes in *A. veronii* HM21 that are important for utilizing heme-containing molecules. We first queried the genome of *A. veronii* for heme utilization genes, which revealed a heme receptor that has an 80% amino acid identity to *A. hydrophila*'s putative outer-membrane heme receptor, *hgpB*. The corresponding open reading frame is 2106 bp long and is flanked by ORFs encoding an uncharacterized iron-regulated protein upstream and a transcriptional regulator downstream (Figure 2A).

Bioinformatic analyses provided further support for the role of the *hgpB* homolog in heme utilization. A PDB (Protein databank) analysis revealed a significant similarity (*e*-value 1.5 × 10^−36^) to ShuU, the crystallized heme/hemoglobin outer membrane transporter from *Shigella dysenteriae*, which forms a beta barrel structure (Cobessi et al., 2010). InterProScan further supported this by revealing the presence of a signal sequences, TonB-dependent receptor plug and a beta- barrel. SignalP analysis indicated the presence of a signal sequences with a cleavage site between residues 24 and 25. This information supports the role of this protein as a putative heme receptor and in turn we name this gene *hgpB*.

We then generated mutants within the chromosome of *A. veronii* by recombining a fragment of DNA containing a portion of *hgpB* fragmented by an antibiotic maker into HM21RS and S-497S yielding mutants with an interrupted *hgpB* (H-890, heme mutant) and a double mutant (SH-894, siderophore-heme double mutant). The interruption of *hgpB* was verified by PCR and the expected band shift was observed in the mutants but not the parent strains (data not shown), verifying that the Cm^R^ cassette interrupted *hgpB*.

*In vitro* phenotypes of heme utilization mutants were examined under iron limiting conditions by measuring growth yields and plating on LIM EDDA + heme plates (Figure 2B and Supplementary Figure 1). In LIM, HM21R, H-890, S-497, and SH-894 reached a similar density 24 h after inoculation (Figure 2B). In LIM containing 150 μM EDDA, HM21R and H-890 had similar growth yields, suggesting that *hgpB* is not essential for growth in the presence of EDDA and that iron-siderophore utilization is sufficient. As expected, the growth yield of SH-894 was significantly lower than HM21R when grown in LIM containing 150 μM EDDA, showing the same phenotype as S-497. The addition of heme (50 μM) did not restore growth of SH-894 to parent strain levels, indicating that SH-894 lost the ability to utilize iron through both siderophores and heme utilization (Figure 2). These results confirmed *hgpB* is required for utilization of iron bound to heme. When grown on LIM EDDA + heme plates similar results to the growth yield experiments in liquid media were obtained. HM21R, H-890, S-497 grew similarly on the plate while SH-894 did not grow on the plates indicating the loss of ability to utilize heme-containing molecules (Supplementary Figure 1).

### The ability to utilize heme-containing molecules is important for colonization of the leech crop

**Figure 3:**
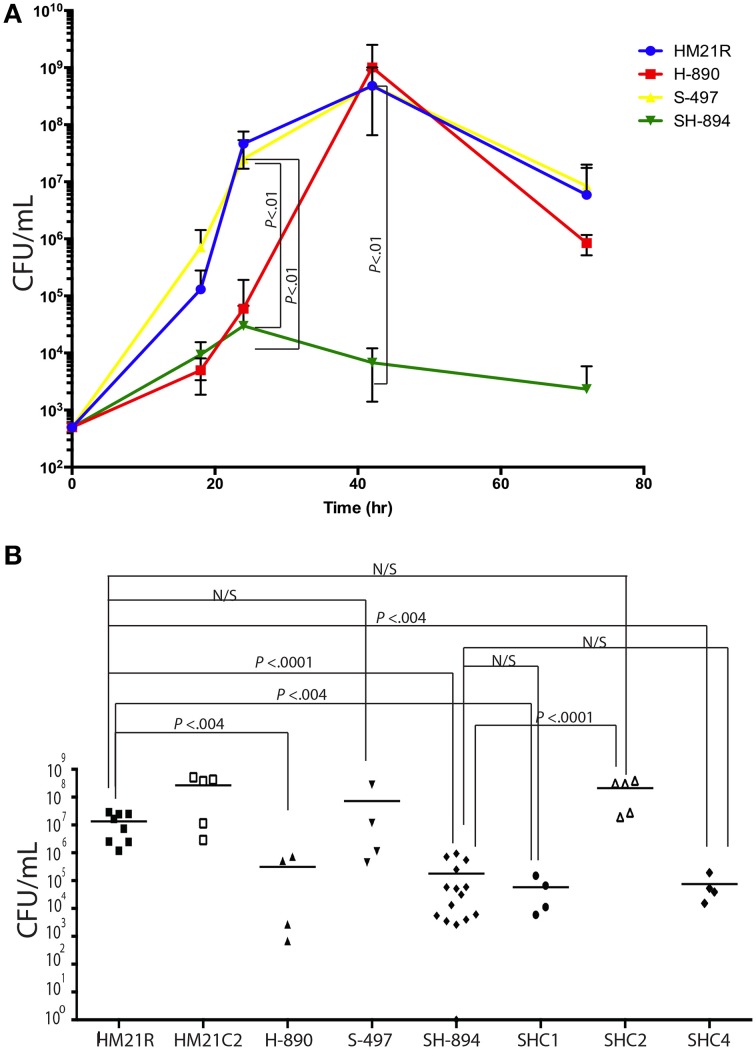
***In vitro* and *in vivo* proliferation of iron utilization mutants**. A time course of growth in blood of Hm21R (blue); H-890 (red), the *hgpB* mutant; S-497 (yellow), the siderophore mutant; and SH-894 (green), the double mutant at 18, 24, 42, and 72 h is shown **(A)**. This revealed that in blood either *viuB* or *hgpB* suffice for growth. The symbols represent the average of 3 leech gut samples **(A)**. Colonization of iron utilization mutants and complemented strains within the leech crop is shown **(B)**. Each symbol represents the colonization level in one leech. The statistically significant difference between the parent strain and each mutant was performed using a Mann–Whitney test. Bonferroni corrections were preformed and the critical threshold is 0.007. N/S, not significant.

Next we examined the ability of mutants to grow in blood as a control over a 72 h time course (18, 24, 42, and 72 h) (Figure 3A). 18 h after inoculation, HM21, H-890, S-497, and SH-894 grew to similar levels. By 24 h, H-890 and SH-894 had a significantly lower CFU/mL when compared to the parent strain, HM21 and S-497. These data suggest that at 24 h the heme receptor is more important for iron acquisition in blood than siderophores. By 42 h after inoculation, SH-894 had a growth defect when compared to the parent strain but for H-890 growth is restored, suggesting that siderophores can compensate for the heme-utilization defect 42 h after inoculation *in vitro*. By 72 h, the concentration of CFU decreased in all strains, analogous to a decrease observed during late stationary phase.

The ability of the mutants to colonize the digestive tract of *H. verbana* was monitored at 24 h (Figure 3B) by introducing the bacteria in a blood meal. H-890 and SH-894 colonized the leech to a significantly lower level then the parent strain and S-497 (Whitney test, *P* = 0.0159) indicating that the ability to utilize heme containing molecules, rather than relying on siderophores for obtaining iron, is necessary for *A. veronii* to acquire iron inside the crop and successfully colonize the leech.

### Complementation of heme utilization mutant SH-894

As the goal of our investigation was to determine which iron utilization system was required for colonizing the leech digestive tract, we focused on complementing the *hgpB* locus and evaluated its role in utilizing heme as an iron source and in the colonization of the leech in the mutant (SH-894) with the inactivated *hgpB* locus. *hgpB* was introduced to SH-894 on a Tn*7*, yielding SHC1 (Table 1). Complementation was initially assessed by growth curves in LIM, LIM containing 150 μM EDDA, and LIM containing 150 μM EDDA supplemented with heme (50 μM). As expected, SHC1 grew to similar rates as the parent strain in LIM suggesting no general growth defect (data not shown). Since the complemented strain still retains the original loss of siderophore utilization, 150 μM of EDDA interfered with the growth of SHC1 but not with the growth of HM21, which is consistent with above findings from S-497 (Siderophore mutant) (data not shown and Figure 1). The addition of heme relieved the growth inhibition of SHC1 but only after a very long lag phase (Supplementary Figure 2). These data link the *hgpB* mutation to the heme utilization phenotype as *hgpB* restored the ability to utilize heme *in vitro* when EDDA is present, however, the strain had a long lag phase suggesting a slow adaptation to a new source of iron. Leech colonization assays revealed that SHC1 was unable to grow or colonize to equal levels as the parent strain (Figure 3B). These data suggest that complementing SH-894 with *hgpB* alone did not restore the ability of the mutant to colonize the leech gut.

One hundred and seventy-nine bp downstream of *hgpB* is another open reading frame, ORF3, that is transcribed in the same direction and predicted to encode a transcriptional regulator (Figure 2A). Using ProDom, a comparison of the deduced amino acid sequence was done suggesting that the protein belongs to the LuxR family of regulators, possesses both the DNA-binding and autoinducer-binding regions (Corpet et al., 2000; Aziz et al., 2008) and 39.2% of the amino acids were identical to the verified LuxR homolog from *A. hydrophila*, AhyR. Querying the HM21 genome with AhyR revealed the presence of two additional homologs. One of the other homologs has the greatest sequence identity to AhyR (91.2%) and is found next to the LuxI homolog, which is the typical gene organization in *Aeromonas* species (Kirke et al., 2004).

We examined the role of ORF3, which encodes the regulator, and the intervening DNA region of *hgpB*, by constructing a Tn*7* vector carrying both *hgpB* and ORF3. This construct was introduced in the double mutant, SH-894 yielding SHC2. In contrast to SHC1, SHC2 grew without a significant growth lag in LIM supplemented with EDDA and heme (Supplementary Figure 2). These data suggests that ORF3 alleviated the long lag phase. Based on the alleviation in the lag phase when growing on heme and the similarity of ORF3 to LuxR-type regulators, we propose to name this gene *hgpR*, for regulator. Complementation of the leech colonization phenotypes was assessed using SHC2 at 24 h (Figure 3B). The siderophore-heme receptor double mutant, SH-894, grew to similar levels as the parent strain, Hm21R, when complemented with a Tn*7* carrying both *hgpB* and *hgpR*. This links the colonization defect to the heme utilization locus.

One explanation of these results is that the mutation in *hgpB* could have caused a polar mutation, reducing or preventing the transcription of *hgpR*. RT-PCR was done to detect expression of *hgpB* and *hgpR* in strains HM21R, SH-894, SHC1, and SHC2 grown in LIM for 24 h with heme to determine whether there was a polar effect. The positive control transcript, *rpoB*, was detected in all strains (Data not shown). As expected, *hgpB* transcripts were detected in HM21R, SHC1, and SHC2 but not in SH-894, suggesting that *hgpB* was complemented in SHC1 and SHC2 (Supplementary Figure 3). RT-PCR of *hgpR* revealed its transcript was detected in HM21R and SHC2 but not in SHC1and SH-894 (Supplementary Figure 3). An alternative explanation is that untranslated regions, UTRs, of the mRNA were responsible. A third complemented strain was constructed that included the intergenic region up and downstream of *hgpB*, SHC3 (Supplementary Figure 4). These data show that the intergentic regions and *hgpB* could not restore the colonization defect within the leech crop. While it is still possible that the UTR is larger than the intragenic regions, the data as a whole provide support for a polar effect of the *hgpB* inactivation on *hgpR*.

**Figure 4:**
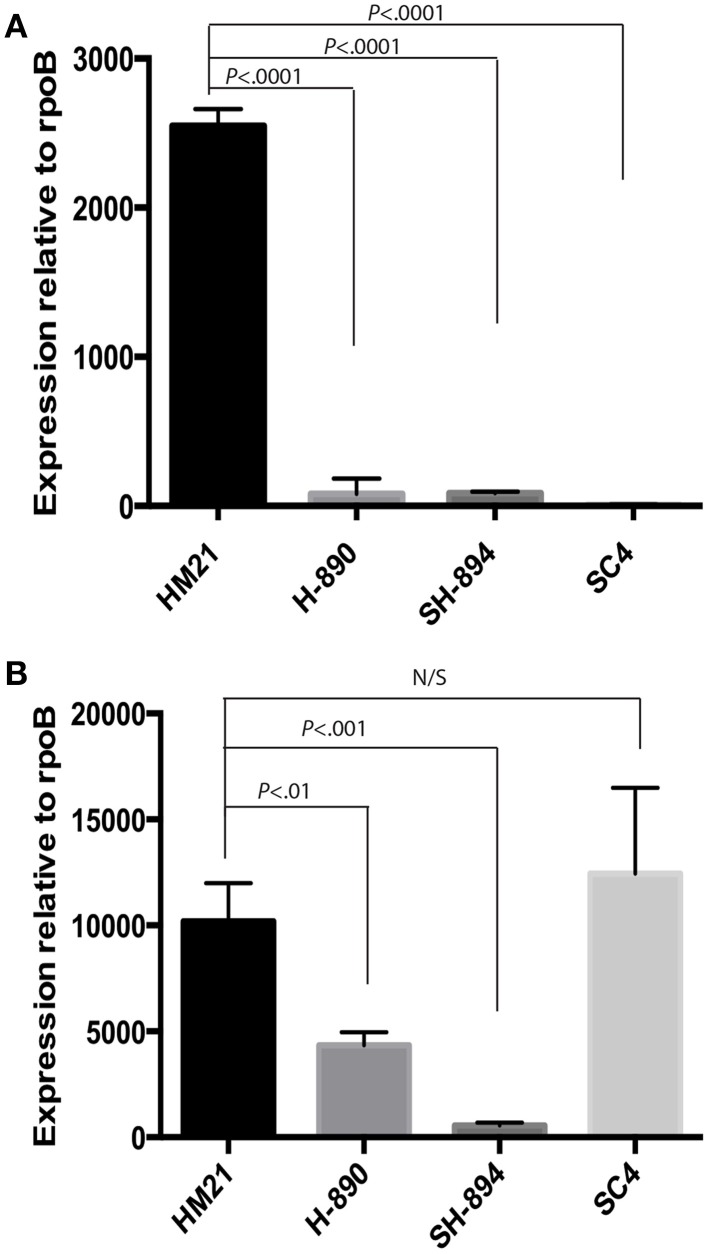
**QRT-PCR on *hgpB* mutants**. Transcripts from cultures growth in an iron limiting medium with heme present was investigated for expression of *hgpB* **(A)** and *hgpR* **(B)** relative to *rpoB*. As expected the expression of *hgpB* is significantly lower in both *hgpB* mutants then HM21, indicating that the gene was successfully knocked out **(A)**. The expression of *hgpR* is significantly lower in both *hgpB* mutants when compared to HM21, revealing a possible polar mutation effecting transcription of *hgpR* **(B)**. Complementation with only *hgpR* (SHC4) did not restore expression of *hgpB*, providing further evidence for a polar mutation **(A)**. The statistically significant difference was determined using *P* < 0.05 Mann–Whitney test. Bonferroni corrections were preformed and the critical threshold is 0.016. Relative expression was calculated using the 2^−ΔCt^ method.

We wanted to determine whether the observed defects were due directly to the downstream effect on *hgpR*. For this goal, we complemented SH-894 with *hgpR* only, yielding SHC4. We then tested the ability of SHC4 to grown on LIM EDDA + heme plates (Supplementary Figure 1 EDDA). SHC4 was unable to grow on LIM EDDA + heme indicating that the heme utilization phenotype was not complemented. We also tested the ability of SHC4 to colonize the leech crop (Figure 3B). SHC4 was unable to colonize to similar levels as the parent strain. To investigate whether *hgpR* was being expressed in SHC4 we did qRT-PCR on *in vitro* cultures (Figure 4). We took overnight cultures of HM21, H-890, SH-894, and SHC4 grown in LIM + HEME and extracted RNA. After reverse transcribing the mRNA into cDNA we determined the expression of *hgpB* (Figure 4A) and *hgpR* (Figure 4B) relative to the house keeping gene *rpoB*. As expected, the expression of *hgpB* in H-890, SH-894 and SHC4 was significantly lower than in HM21 (Figure 4A). The expression of *hgpR* in H-890 and SH-894 was significantly lower than in HM21 but as expected in SHC4 the expression of *hgpR* was similar to HM21, indicating SHC4 had been complemented with *hgpR* and this gene was being expressed. Together these data show that *hgpR* alone is unable to restore the leech colonization defect and that *hgpB* and *hgpR* are both important for leech colonization.

### Restoration of heme utilization phenotype using free iron

**Figure 5:**
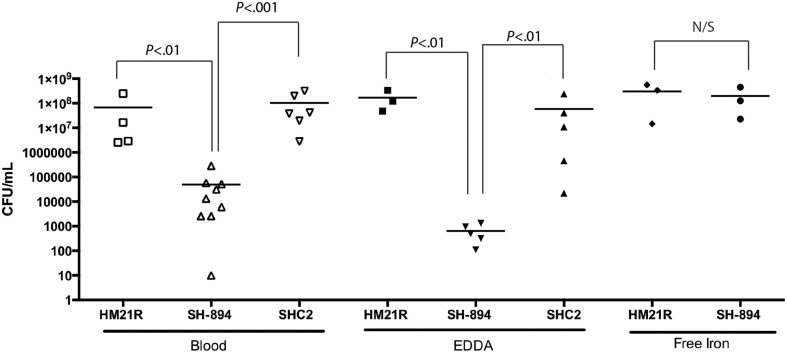
**Restoration of double mutant (SH-894) using free iron**. The leech crop colonization of the double mutant using a chemically manipulated blood meal is shown. One hundred and fifty micrometer EDDA was added to the blood meal along with strains to show minimal amounts of a non-protein-bound iron (free iron) with in the leech crop. SH-894 cannot colonize the leech crop. When 160 μM of ferric chloride (free iron) is added to the blood meal the colonization defect of the double mutant was restored, further linking the inability to obtain iron within the leech crop. Each symbol represents the colonization level in one leech. The statistically significant difference between the parent strain and each mutant was performed using a Mann–Whitney test. Bonferroni corrections were preformed and the critical threshold is 0.025.

We wanted to test if there is a link between the inability to utilize iron from heme-containing molecules and the colonization phenotype (Figure 5). Leeches were fed blood meals containing either HM21 or SH-894 supplemented with EDDA (150 μM) or ferric chloride (160 μM). When inoculated into the blood meal containing EDDA, SH-894 growth did not change when compared to growth in blood suggesting that there are not sufficient amounts of free iron present in the blood meal or leech crop to allow the growth of SH-894. When ferric chloride was added to the blood meal SH-894 grew to similar levels as the parent strain while in animals fed a normal blood meal SH-894 failed to establish itself. These results suggest there are insufficient amounts of free iron in the crop to support *Aeromonas* growth unless iron can be liberated from heme-containing molecules and that this accounts for the colonization defect of the *hgpB* mutant in the leech crop.

#### Presence of iron uptake genes in *aeromonas* species and among *A. veronii* strains

The importance of iron utilization in this beneficial symbiosis and the role of iron as a virulence factor in other organisms led us to investigate the distribution of iron-uptake genes among 67 genomes representing 28 *Aeromonas* species (Table 2). This set of strains consists of isolates obtained from a wide range of samples including clinical, veterinary and environmental. All genomes except *A. simiae* CIP107798^T^ contained the heme receptor, *hgpB*, indicating that the ability to obtain iron from heme is widespread among members of this genus. In contrast the distribution of *viuB*, 2,3-dihydroxybenzonate AMP ligase, and amonabactin was not uniform. The 2,3-dihydroxybenzonate AMP ligase that is involved in the biosynthesis of enterobactin was present in all 11 *A. veronii* genomes but absent in all 4 *A. hydrophila* and 2 *A. caviae* genomes. In contrast, all *A. hydrophila* and *A. caviae* encoded for *amoA* that is involved in the biosynthesis of amonabactin, while none of the *A. veronii* strains encoded for AmoA. The other iron utilization genes were more uniformly distributed. All genomes encoded a ferrous iron transport system. While siderophore transport permease, enterobactin receptor, ABC-type Fe3+ transport system, iron III dicitrate transport system and ferric hydroxamate ABC- transporters were present in the vast majority of the genomes. It is important to point out that the absence of a hit does not necessarily imply the absence of the gene. For example, the iron uptake regulator, Fur, was missing from *A*. sp. 159 this is probably due to low sequence similarity or an incomplete genome as this regulator would be predicted to be present in all of the genomes. These data indicate that heme utilization may be a molecular requirement for both pathogenic and beneficial relationships. 34 out of 67 strains have a similar *viuB* as HM21 indicating the importance of the ability to utilize iron from a siderophore, even when the bacteria did not posses the genes to produce their own siderophore.

**Table 2:** **Nucleotide percentage identity to iron uptake genes in *Aeromonas* species and among *A. veronii* strains**.

	**Ferric_uptake_regulation_ protein_FUR**	**Heme_receptor**	**ABC-type_Fe+_Siderophore_transport_system,_permease_**	**viuB**	**entE (2,3-dihydroxybenzoate-AMP ligase)**	***A. hydrophila* amonabactin (amoA)**	**irg_enterobactin_receptor_Aeromonas hydrophila subsp**.	**ABC-type Fe3+ transport system**	**Ferrous_iron_transport_protein_A**	**Ferrous_iron_transport_protein_B**	**lron_lll_dicitrate_transport_system**	**lron_dicitrate_transport_system_fecB**	**Ferric_hydroxamate_outer_membrane_receptor_FhuA**	**Ferric_hydroxamate_ABC_transporter permease_component_FhuB**	**Ferric_hydroxamate_ABC_transporter,_ATP-binding_p rotein_FhuC**	**Ferric_hydroxamate_ABC_transporter periplasmic_FhuD**	**hgpR**
A_allosaccharophila_BVH88	98.1	96.9	92.5	94.4			81.2	94.2	93.8	97.7	97.4	97.4	95.7	94.6	95.4	95.7	
A_allosaccharophila_CECT419	99.1	94.5	93.5	95.7			85.1	96.2	100	97.1	97	96.3	90.7	93.5	96.2	95.2	
A_aquariorum_AAKl	91.8	82	79.8	75.3	78.8	90.7	91.2	80.3	88.9	88.5	88.9	79.7	88.7	86.3	91.8	83.6	
A_aquariorum_CECT7289T	91.5	82	79.3			90.8	90.9	79.9	88.9	88.4	88.9	80.3	88.5	86.4	91.5	83.9	75.4
A_austrailiensis_CECT8023T	97.4	92.3	94.7	95.9	94.74		82.9	94.9	90.8	91.5	95.9	95		83.6	77.8		
A_bestarium_CECT4227T	91.1	82.5	79.2		76.78	84.5	92.9	79.6	87.2	87.2	80.6	78.3	88.4	86.4	90.1	84.4	
A_bivalvium_CECT7113T	87.9	82	79.2			87.8	86.5	80.4	87.5	84.7	95.1	77.9	86.1	84.9	88.5	82.3	
A caviae Ae398	91.3	83	77.8		78.14	89.7		79.5	85.5	82.5	90.4	78.5	84.8	84.3	89.9	78.6	
A_caviae_CECT838T	90.9	83	78		77.78	89.5		79.4	85	82.6	90.4	78.4	84.8	84.3	89.9	78.7	
A_diversa_CECT4254T	87.7	75.5	79.8		82.5	82.3	89.6	78	79.7	78.5	93.2						
A_encheleia_CECT4342T	91.2	83.7	78.6		90	81.3	78.8	79.7	87.2	84.1	94.3	78	85.2	82.5	86	81.5	
A_enteropelogenes_1999	90.4	87.3	88.5	79.5	83.32		83.5	85.2	88.9	87	87.8	81.6	88.1	85.7	90.7	86.3	
A_enteropelogenes_CECT448	90.7	87	87	79.6	83.56		83.5	86.2	88.9	86.3	88.1	81.8	88	85.1	89	86.3	
A_eucrenophila_CECT4224T	90.7	82.9	84.2		86.08	84.1	78.8	80.6	87.7	82.9	87	77.8	85.4	83.8	88.6	80.3	
A_fluvialis_LMG24681T	94.1	93.5							91.6	93.8	85.5		92	93.4	96.9	93.3	
A_hydrophila_CECT839T	91.9	81.4	79.1			92.4	100	79.8	88.9	88.9	90.9	80	89.4	86.9	91.9	84.2	
AJiydrophila_CIP107985	91.9	81.6	78.7		81.69	92.9	98.4	80.7	88.9	88.7	86.2	79.6	88.7	85.7	90.6	83.2	
A_hydrophila_SNUFPCA8	91.5	81.6	78.9		77.38	92.8	97.6	79.9	89.8	88.8	94.3	79.5	89.4	86.3	91.7	84	
A_hydrophila_ML09119	91.7	81.4	78.8		76.42	92.7	98.1	80.7	88.5	88.5	90.9	79.7	88.9	85.7	92	84.4	
A_dhakensis_014	91.5	81.9	79.3			91.5	91.1	80.6	89.8	88.5	88.9	79.3	88.8	86.2	90.9	83.2	75.8
A_dhakensis_116	91.5	81.9	79.3			91.5	91.1	80.6	89.8	88.5	88.9	79.3	88.8	86.2	90.9	83.2	75.8
A_hydrophila_145	92	81.7	79.4			91.2	91.3	79.2	87.6	88.6	88.9	79.4	88.6	85.9	91.5	84	75.7
A_dhakensis_173	92	82.1	79.4	75 1		92.2	91	79.3	88.9	88.1	88.9	79.8	88.8	86.8	91.5	83.7	
A_dhakensis_187	92	82.4	79	75.7	76.42	91.3	91.2	79.6	88.5	88.5	88.9	79.5	86.5	86.1	91.6	84.3	76.6
A_hydrophila_226	91.5	81.8	78.5			93.2	97.9	79.9	88.1	88.8	90.9	79.6	89.1	86.6	91.9	83.1	
A_dhakensis_259	92	82	79.4	75.5	76.06	91.3	91.1	79.3	88.5	88.5	92.9	79.9	88.7	86.4	91.5	83.4	75.7
A_dhakensis_277	92	81.8	79.5	75		91.4	91.2	79.6	88.9	88.3	88.9	79.3	88.6	86.1	91.3	83.8	76.1
A_hydrophila_289	91.7	81.7	79.1		75	93.7	98.2	79.5	88.9	88.9	94.3	80.1	89.1	86.1	92	83.5	
A_dhakensis_CIP107500	92	82.1	78.4	75.3	76.81	91.3	91.4	80.9	87.6	88.7	88.9	80	86.4	86.4	91.9	84.1	75.6
A_hydrophila_NFl	91.7	81.5	79.4			93.7	98.1	80.4	88.9	88.9	94.3	80.1	89.1	88	92.2	83	
A_hydrophila_NF2	91.5	81.7	79			92.6	98.2	80	88.9	89.1	88.6	79.3	89.1	86.8	91.3	84.5	
A_hydrophila_SSU	92	81.9	79.2	75.5	75.83	91.1	91.1	80.4	88.1	88.2	88.9	80	88.7	86.3	92	82.8	75.8
A_hydrophila_AH4	90.5	82.7	79			92.6	91.5	79.5	87.2	87.3	80.7	77.9	88.1	87.1	87.2	84.6	
AJandaei_CECT4228T	92.9	89.7	95	84.4	93.89		82.4	94.8	93.4	88.6	96.8	89.8		93	77.9		77.2
A_media_CECT4232T	91.4	82.9	78.7		76.13	88	87.2	79.7	86.7	82.9	92.7	78.1	86.1	84.1	87.9	80.4	
A_media_WS.42948	91.4	84.2	78.5			88.1		79.7	86.7	82.9	96.9	78.4	86.8	84.2	89.5	80.8	
A_mollusconjm_CIP108876T	87	77.7	79.4			88.8	82.4	78.6	79.8	80.9	90.2	75.4	83.6	77	87.2		
A_piscicola_LMG24783T	92	83.1	79.6		77.83	85.4	91.8	79.1	87.2	87.3	80.7	78.5	88.1	87	89.3	82.9	77
A_popoffii_CIP105493T	89.7	81.3	79.6		76.19	83.8	84	79.6	91.6	88.6	80.2	85.9		90.1	83.9		78
A_rivuli_DSM22539T	88.1	78.4	79.3	82.6	91.23	80.5	82	78.4	80.6	81.3	80.6	78.6	84.8		80.3		
A_salmonicida_01B526	91.3	82.2	79.2	83.9	75.59	100	89.5	78.4	85.4	86.2	92.1	78.1	87.8	85.3	89.1	82.6	
A_salmonicida_34	90.5	82.4	79.3			85.5	89.7	79.5	85.8	86.2	90.7	78.2	88	85.2	89.6	82.1	79.8
A_salmonicida_A449	91.6	82.2	79.2		75.59	100	88.8	78.4	85.4	86.2	92.1	78.1	87.8	85.3	89.1	82.6	
A salmonicida A503	91.6	82.5	79.2		75.4	85	88.9	78.4	85.4	86	92.1	78.1	88	85.4	89.3	82.7	
A_salmonicida_CIP103209T	91.6	82.2	79.2		75.59	100	89.2	78.4	85.4	86.2	92.1	78.1	87.8	85.3	89.1	82.6	
A_sanarelli_LMG24682T	91.6	82.4	77.6		76.53	94.4		79.9	87.2	82.4	92.7	78.4	86.1	82.4	87.2	79.4	
A_sch u berti i_CECT4240T	88.8	76.1	78.6			78.6	79	79.3	80	77.4	88.6						
A_simiae_CIP107798T	86.8		77				81.2	78.7	78.7	79.2	90.7	78.2	79.7		76.8		
A_sobria_CECT4245T	96	91.7	93.6	95			81.2	96.3	92.9	91	95.5	95.9	95.9	92.8	95.7	93.5	81.9
A_sp_159	91.8	85	94.6	96.9			80	95.2	99.6	97	96.1	96.6	91.2	94.6	96.9	95.7	97.8
A_sp_MDS8		81.9	78.8	75.1		90.8	91.3	80.5	87.6	88.5	88.9	79.5	88.4	85.8	91.6	84.7	
A_taiwanensis_LMG24683T	92.3	100	91.5	97.3	95.39	83.9	85.2	95.7	86.3	82.9	87	78.2	85.4	93.5	89.2	79	97.6
A_tecta_CECT7082T	91.4	82.8	79		76.37	80.8	85.6	79.6	87.2	83.4	79.9	77.4	85.3	82.8	86.4	79.7	
A_trota_CECT4255T	90.8	87.6	86.9	79.5	82.97		83.5	85.1	88.9	86.1	87.6	82.1	87.8	85.2	89.3	86.3	
A_veronii_AER28	99.1	97.4	94	96.6	95.08		82.8	96.2	99.6	97.3	97.6	97.5	97.7	94.2	96.7	95.5	
A_veronii_AER39	100	98.1	94.1	97.2	95.45		81.3	96.6	99.6	97.2	96.8	96.7	97.4	94.6	96.5	95.1	
A_veronii_AER397	98.4	97.5	90.4	97.6	95.02		81.3	95.7	99.6	97.3	95.2	90.8	97.5	96.8	96.3	94.7	
A_veronii_AMC34	98.6	95.9	94.3	94.2	95.69		81.2	96.3	92	97	96.6	96.9	93.4	95.4	96.3	95.8	87.6
A_veronii_AlVlC35	99.3	96.9	90.2	97.7	93.83		81.3	96.2	99.6	97.5	97	96.6	97.8	93.9	97.1	95.6	
A_veronii_8565	98.4	97.5	90.4	97.6	95.02		81.3	95.7	99.6	97.3	95.2	90.8	97.5	96.8	96.3	94.7	
A_veronii_CECT4257T	98.4	97.5	90.4	97.6	95.02		81.3	95.7	99.6	97.3	95.2	90.8	97.5	96.8	96.3	94.7	
A_veronii_Hm21	100	100	100	100	100		80.4	100	100	100	100	100	100	100	100	100	100
A_veronii_LMG13067	99.3	97.3	95.5	97	93.59		86.1	96.1	99.6	97.3	97.6	97.7	97.6	95.5	96.3	95.5	
A_ichthiosmia_CECT4486	99.1	97	94.5	97.4	95.75		80.9	95.5	99.6	97.4	94.1	96.9	89.5	94.7	96.7	96.3	97.6
A_culicicola_CIP107763	99.1	84.7	94.1	97.7	95.32		82.8	94.4	99.6	97.6	95.2	96.1	89.6	94.2	96.3	95.4	
	75	80	85	90	95	100											

*Nucleotide percentage identity. The redder the color the closer to 100% identity*.

## Discussion

The primary goal of this study was to investigate the importance of iron-utilization by *A. veronii* in the leech symbiosis using molecular genetic approaches and leveraging access to the genomes of Aeromonads. Because relatively little is known about the genetic requirements for iron-uptake in *Aeromonas*, we used a bioinformatic approach to gain insight into the prevalence of iron utilization genes in this genus (Najimi et al., 2008; Funahashi et al., 2013). Our data reveals that the ability to obtain heme-bound iron is essential for *A. veronii* strain HM21 to successfully colonize the leech crop and that *hgpB* is widespread among Aeromonads.

Focusing initially on siderophores, we were able to show that HM21 synthesizes siderophores and that siderophore utilization is not required for colonizing the leech digestive tract. Similarly, *Photorhabdus luminescens* was shown not to require siderophores to colonize its symbiotic nematode host, *Heterorhabditis bacteriophora*, nor for virulence in the insect hosts (Ciche et al., 2003). This is in contrast to the *V. fischeri* symbiosis where the production of siderophores are essential for colonization of the light organ within the Hawaiian bobtail squid, *Euprymna scolopes* (Graf and Ruby, 2000). In the shipworm symbiosis (another marine system), siderophore biosynthetic genes were found in the genome of endosymbiont *Teredinibacter turnerae* and the siderophore was detected in the shipworm extracts, suggesting that its expression plays a role in this symbiosis (Han et al., 2013). There does not appear to be a strict requirement for siderophore-dependent iron utilization during colonization of animal hosts within beneficial associations, suggesting that alternative mechanism are employed.

In this study, we identified a second distinct iron-utilization mechanism in *A. veronii* strain HM21 involving heme. A potential heme utilization gene, *hgpB* was detected in the HM21 genome. *hgpB* mutants were constructed to characterize the role of *hgpB* in iron uptake from heme and the colonization of the leech. The loss of the ability to acquire iron bound to heme was verified in the double mutant (the *hgpB* and *viuB* mutant, SH-894) when grown in LIM with EDDA + heme, which supported the importance of *hgpB* in utilization of iron bond to heme. Bacterial growth data from a time course performed in blood indicated that either iron acquired by a siderophore or heme receptor is sufficient for *A. veronii* to proliferate in a blood environment. The double mutant grew to significantly lower concentrations in blood when compared to the parent strain and the other mutants. While some bacteria, including HM21, have other iron acquisition system, including ferrous and ferric iron receptors (Table 2), this data suggests that for growth in blood these other iron utilization systems are not sufficient for HM21 to proliferate.

Leech colonization assays using the *hgpB* mutants revealed that the ability to acquire iron bound to heme is essential for *A. veronii* to colonize the leech crop. These data also suggests that iron potentially bound to transferrin is not present at sufficient levels, cannot be utilized by *A. veronii* or that other bacteria such as the abundant *M. hirudinis* outcompete *A. veronii* for transferrin. Utilization of iron bond to heme necessitates access to hemoglobin that is contained within the erythrocytes. While early studies reported that the erythrocytes remain physically intact for many months inside the crop of the leech and do not fully lyse until they enter the intestinum (Graf, 1999b), we have since demonstrated that the ability of *A. veronii* to lyse erythrocytes in the leech crop is essential for initial colonization (Maltz and Graf, 2011). The ability to access heme-bound iron provides further mechanistic evidence that at least a portion of the erythrocytes are lysed or are being permeabilized by *A. veronii* in the leech crop. Whether this aids the leech in digestion of the consumed blood meal or competes with the host for nutrients remains to be evaluated.

Complementation of the *hgpB* mutants indicated that *hgpB* alone was not sufficient to restore the leech colonization phenotype and revealed the requirement for both *hgpB and hgpR* to be present in order to restore the colonization ability of the double mutant. RT-PCR and qRT-PCR data revealed a possible polar effect of the *hgpB* mutation resulting in a reduced expression of *hgpR*. Although the two genes are separated by 336 bp, the insertion of a large antibiotic cassette insertion could cause a frameshift downstream. Another possibility is that the fragment of *hgpB* cloned for complementation (SCH1) is missing a critical 3′ UTR element that is needed for transcript stability. This is unlikely since SHC3 (*hgpB* + up and downstream intergenic region) did not complement the leech defect. Our data suggest that *hgpB* and *hgpR* are important for iron utilization bound to heme and which in turn is important for leech colonization. Future studies will investigate the role the regulator (*hgpR*) plays on *hgpB* and possibly other genes by making clean mutations in this heme utilization region.

While heme utilization has been mostly characterized in pathogenic associations, in two other beneficial symbioses, heme utilization has been shown to be important. In the Tsetse fly symbiont, *Sodalis glossinidius* analysis revealed that a heme receptor is upregulated inside the host digestive tract and that this heme receptor is important for colonization (Hrusa et al., 2015). As this is also a blood-feeding host and the symbiont can reside inside the digestive tract, which is an analogous habitat to the leech. However, in the *V. fischeri-E. scolopes* symbiosis where siderophores are required for symbiotic competence, a heme uptake cluster is also important for colonization for the light organ (Septer et al., 2011). In this case it was proposed that blebs of epithelial cells that might contain heme enter the light organ space and could serve as the source of iron.

The analysis of the *Aeromonas* genomes for iron utilization genes revealed that most *Aeromonas* strains contain a wide range of iron utilization genes. Interesting, the heme receptor is present in all but one of the genomes analyzed even though only *A. hydrophila, A. jandaei*, and *A. veronii* strains have been isolated from leeches (Silver and Graf, 2009; Colston et al., 2014). These data suggests a role for the heme receptor in other habitats, including *Aeromonas* infections in humans, fish and amphibians. The heme receptor has also been shown to be dispersed in other species of bacteria with a wide host range, for example the Vibrios i.e.: *Vibrio. anguillarum* (a fish pathogen), *V. cholerae* (human pathogen), *V. fischeri* (beneficial symbiont) *V. mimicus* (human pathogen), and *V. vulnificus* (human pathogen) (O'Malley et al., 1999; Mouriño et al., 2005; Runyen-Janecky, 2013). Like Aeromonads, Vibrios can be found in different states, environmental, pathogenic, and symbiotic, indicating a role for the heme receptor in multiple habitants in multiple genera of bacteria.

Coexistence with a host requires a precise balance, allowing bacterial growth while preventing overgrowth. In animals with specialized symbiotic organs this can be achieved by controlling the release of nutrients, but in a digestive tract the control mechanisms probably require additional layers as the ingested food provides nutrients to both host and microbes. One such mechanism can be the restriction of free iron. There are additional requirements a microbe must posses to overcome physical, cellular, and molecular barriers presented by the host. As scientists investigate these bacterial interactions, it is becoming clearer that a number of these molecular requirements for microorganisms to colonize animals are similar between pathogenic and mutualistic associations, despite the different outcomes (Hentschel et al., 2000; Ochman et al., 2010). Iron is a nutritional requirement for most bacteria. Inside a host, protein binding sequesters iron. Therefore, for bacteria to overcome this barrier within a host they must acquire iron bound to host proteins. In a pathogenic state, it has been shown that bacteria require iron not only as a nutritional source but can also sometimes sense iron limitation with a host environment and release bacterial toxins or virulence factors (Litwin and Calderwood, 1993). Examples of iron-regulated virulence factors include; Shiga toxin (*Shigella dysenteriae* Type I), Exotoxin A *(Pseudomonas aeruginosa*), Diphtheria toxin (*C. diphtheria*) and SLT-I (*E. coli*) (Litwin and Calderwood, 1993). Our findings suggest, that within this mutualistic relationship, a microbe with a nutritional requirement for iron must posses a high-affinity iron utilization system to proliferate in the leech crop. These data indicate the importance of utilizing iron bond to heme for *A. veronii* in the colonization of the leech gut. We also show within the *Aeromonas* genus heme utilization is not limited to mutualistic associations since the presence of *hgpB* was found in both clinical and environmental isolates, further confirming the interesting parallelism between pathogenic and mutualistic relationships.

### Conflict of interest statement

The authors declare that the research was conducted in the absence of any commercial or financial relationships that could be construed as a potential conflict of interest.
